# Antihyperuricemic Effect of *Dendropanax morbifera* Leaf Extract in Rodent Models

**DOI:** 10.1155/2021/3732317

**Published:** 2021-07-23

**Authors:** Dongho Lee, Jin-Kyoung Kim, Yongjae Han, Kwang Il Park

**Affiliations:** ^1^Department of Anesthesiology and Pain Medicine, College of Medicine, Inje University, Gimhae 47392, Republic of Korea; ^2^Department of Preventive Dentistry, School of Dentistry Kyungpook National University, Daegu 41940, Republic of Korea; ^3^Department of Dental Hygiene, Daegu Health College, Daegu 41453, Republic of Korea; ^4^Institute of Animal Medicine and College of Veterinary Medicine, Gyeongsang National University, Jinju 52728, Republic of Korea

## Abstract

*Dendropanax morbifera* is a well-known traditional medicine used in China and Korea to treat intestinal disorders, urosis, diuresis, and chronic glomerulonephritis. Hyperuricemia is a metabolic disorder characterized by a high uric acid level in serum due to an imbalance between uric acid production and excretion and causes gout. Recently, the prevalence of hyperuricemia worldwide has been continuously increasing. Xanthine oxidase (XOD) inhibitors (allopurinol (ALP) and febuxostat) and uricosuric agents (benzbromarone and probenecid) are used to treat hyperuricemia clinically. However, because these drugs are poorly tolerated and cause side effects, such as kidney diseases, hepatotoxicity, gastrointestinal symptoms, and hypersensitivity syndrome, only a limited number of drugs are available. We investigated the antihyperuricemic effects of *Dendropanax morbifera* leaf ethanol extract (DMLE) and its underlying mechanisms of action through *in vitro* and *in vivo* studies. We evaluated uric acid levels in serum and urine, and xanthine oxidase (XOD) inhibition activity in the serum and liver tissue of a hyperuricemic rat model of potassium oxonate (PO)-induced hyperuricemic rats. In vitro study, XOD-inhibitory activity was the lowest among the test substances at the IC50 of ALP. However, the IC50 of DMLE-70 was significantly low compared with that of other DMLEs (*p* < 0.05). In PO-induced hyperuricemic rats, uric acid (UA) levels in serum and urine were significantly reduced in all DMLE-70 and allopurinol-treated (ALT) groups than in the PC group (*p* < 0.05). UA levels in urine were lower than those in serum in all DME groups. In PO-induced hyperuricemic rats, DMEE-200 reduced UA concentration in serum and increased UA excretion in the urine. These findings suggest that DMLE exerts antihyperuricemic and uricosuric effects on promoting UA excretion by enhanced secretion and inhibition of UA reabsorption in the kidneys. Thus, DMLE may be a potential treatment for hyperuricemia and gout.

## 1. Introduction

Hyperuricemia means elevated uric acid (UA) level (more than 6.8 mg/dL) in the blood [1]. The disease is associated with a significantly increased risk of gout, cardiovascular disease, chronic kidney disease, and type 2 diabetes mellitus [2]. Serum UA (SUA) is the final product of purine metabolism [3]. Approximately two-thirds of SUA is produced from internal metabolic processes, and the rest is due to a high-purine diet [4]. Approximately 60%–70% of UA from the body is excreted through the kidneys, and the remaining is secreted in biliary secretions and the intestine. It is then further metabolized by gut bacteria in uricolysis [5]. Abnormal UA metabolism and decreased excretion by the kidneys are among the major causes of hyperuricemia [6].

Globally, hyperuricemia prevalence appears to be increasing as it is diagnosed in 5%–30% of the general population [7, 8]. It is also higher in men living in developed countries than women [9]. In the United States, the hyperuricemia prevalence rates are 20.2% in men and 20.0% in women [10]. In the Chinese rural population, the total estimated prevalence of hyperuricemia is 10.24% (12.80% in men and 8.56% in women) [11]. In the general Korean population, the age-standardized prevalence of hyperuricemia is 11.4% (17.0% in men and 5.9% in women) [12]. The progressive increase of hyperuricemia worldwide may be linked to the rising prevalence of overweight and obesity and increased consumption of sugar-sweetened beverages, foods rich in purines, and alcohol [13].

As hyperuricemia results from increased production and decreased excretion, or both, of UA [14], it is crucial to prevent and treat the disorder to regulate the SUA level. UA is produced by xanthine oxidase (XOD), a rate-limiting enzyme that oxidizes hypoxanthine to xanthine, which is subsequently converted to UA [15]. Hence, SUA synthesis and concentration can be affected by XOD enzymatic activity [16]. Therefore, proteins involved in UA production and transport in the kidney may act as important drug targets for treating hyperuricemia.

XOD inhibitors (allopurinol (ALP) and febuxostat) and uricosuric agents (benzbromarone and probenecid) are presently used [17] to clinically treat hyperuricemia. However, these drugs are poorly tolerated and cause side effects, such as kidney diseases, hepatotoxicity, gastrointestinal symptoms, and hypersensitivity syndrome [18]. Therefore, more effective therapeutic agents for hyperuricemia with no adverse effects are needed. In previous studies, new therapeutic methods using herbs were offered to overcome these limitations of drugs for hyperuricemia [19].

*Dendropanax morbifera* H. Lév. (DM) is an evergreen broad-leaved tree of the *Araliaceae* family and is well known as a panacea and wild ginseng tree [9]. DM is an endemic species in Korea and is distributed in the country's southern regions [20]. In previous studies, extracts from roots and stems of DM have antioxidant [21], antibacterial [20], anticancer [22], antidiabetic [23], antiobesitic [9], antihyperglycemic [24], and antiatherogenic [25] properties. DM contains various bioactive compounds, such as triterpenoids, polyacetylene, phenolic substances, _L_-arginine, and *γ*-aminobutyric acid (GABA) [26]. _L_-Arginine is a substrate of nitric oxide (NO) synthesis by NO synthase in almost all cell types. NO regulates glucose metabolism, fatty acids, and amino acids in mammals [27]. Additionally, GABA plays an essential physiological role in regulating cardiovascular function.

Therefore, in this study, the antioxidant and xanthine oxidase (XOD)-inhibitory activity of DM leaves (DML) was evaluated using different solvent extraction conditions (hot water and 30%, 50%, and 70% ethanol). Additionally, the antihyperuricemic effects of DML extracts were investigated in a potassium oxonate (PO)-induced hyperuricemia rat model, and the detailed mechanisms were explored.

## 2. Materials and Methods

### 2.1. Plant Extracts

DML was provided by Hyurim Hwangchil Co., Ltd. (Jinju, Korea), and dried in a shade, crushed, and stored in a refrigerator. The extracts were prepared as described by Kim et al. (2016) with some modifications. Approximately 100 g of the plant powder was extracted with 500 mL of water and 30%, 50%, and 70% ethanol solution for 2 h at 80°C. Subsequently, the solvent was evaporated using a rotary vacuum evaporator at 40°C, freeze-dried, and weighted. Following the method above, the extraction was performed in triplicate for each solvent. The freeze-dried powders of DML ethanol extracts were named DMLEs and stored at 4°C until use.

### 2.2. Analysis of Chlorogenic Acid and Rutin Concentrations in DMLEs

The qualitative and quantitative analyses of chlorogenic acid (CGA) and rutin in DMLEs were conducted using an LC-MS 8050 chromatography system (Shimadzu, Kyoto, Japan) composed of a binary solvent delivery system (LC-30 AD), a controller (CBM 20A), an autosampler (SIL-30A), and a column thermostat (CTO-20AC). Tandem mass spectrometry (MS/MS) analysis was performed positively and negatively on a triple quadrupole equipped with a positive electrospray ionization (+ESI) source. The optimal parameters of ESI-MS were as follows: interface temperature of 340°C, DL temperature of 200°C, nebulizing gas flow at 2.8 L/min, heating gas flow at 8 L/min, and temperature of drying gas at 400°C. The active biological compounds were monitored using a scheduled multiple reaction monitoring mode and separated using a Kinetex C18 column (150 × 2.1 mm, 2.6 *µ*m; Phenomenex, Torrance, CA, USA) at a flow rate of 0.3 mL/min, injection volume of 2 *µ*L, and separation temperature of 40°C. The mobile phase consisted of water containing 5 mM ammonium acetate and 0.1% formic acid (A) and methanol containing 2.5 mM ammonium acetate (B) (gradient 0–18 min; 5%–100% B).

### 2.3. 2,2-Diphenyl-1-picryl-hydrazyl (DPPH) Radical Scavenging Assay

The 2,2-diphenyl-1-picryl-hydrazyl (DPPH) radical scavenging assay was conducted following the description by Okoh et al. using vitamin C as the positive control [28]. Here, 0.1 mL of 0.135 mM DPPH in methanol was mixed with 1.0 mL of the solution prepared in methanol containing 0.025–0.50 mg/mL of DMLEs, CGA, rutin, and vitamin C. The reaction mixture was vortexed thoroughly, left in the dark at 25°C for 30 min, and measured at 517 nm using a spectrophotometer. The DPPH radical scavenging effect was calculated using the equation below:

(1) $$\begin{array}{c} \text{DPPH radical scavenging activity }\left(\%\right)=\left(1-\frac{\text{A}{}_{s}}{\text{A}{}_{c}}\right)\times 100. \end{array}$$

Here, *A*_*c*_ is the absorbance of the control reacted with methanol (50 *μ*L) and DPPH working solution (1 mL); and *A*_*s*_ is the absorbance of the samples.

The sample concentration required for inhibiting 50% DPPH radicals (IC_50_ DPPH values) was obtained by extrapolating the regression analysis. Antioxidant activity was evaluated based on this IC_50_ value.

### 2.4. Measurement of Reactive Oxygen Species (ROS) Production

The acute myelogenous leukemia cell line OCI-AML-2 line from the Ontario Cancer Institute (Toronto, Canada) was cultured at 37°C in a 5% CO_2_ atmosphere using minimum essential medium alpha (*α*-MEM) (Gibco BRL, Grand Island, NY, USA) with 10% heat-inactivated fetal bovine serum. The drug-resistant AML-2 sublines were selected from a parental cell line (AML-2/WT) after chronic exposure to doxorubicin. The cells were finally cultured in a fixed concentration (100 ng/mL) of doxorubicin. The AML-2/DX100 cells were characterized by catalase downregulation and used to determine antioxidant effects [29]. The cells were seeded onto 96-well plates at a density of 1.5 × 10^4^ cells per well in 100 *μ*L of growth media. At 50% confluence, cells were then loaded with 10 *μ*M 2′,7′-dichlorofluorescein diacetate (DCFH-DA; Sigma, St. Louis, MO) and 4 mM hydrogen peroxide (H_2_O_2_; Sigma, St. Louis, MO) in Hanks' balanced salt solution (HBSS; Gibco, Grand Island, NY, USA) at 37°C for 30 min. After loading, cells were washed twice with 200 *μ*L of HBSS to remove excess fluorescent dye. Cells were then treated with 50 and 100 *µ*g/mL of DMLEs, CGA, rutin, and vitamin C and washed twice with 200 *μ*L of HBSS, and 100 *μ*L of HBSS/well was added. The fluorescence intensity of DCFH-DA was measured at an excitation wavelength of 485 nm and an emission wavelength of 520 nm. Cells treated with H_2_O_2_ without DMLEs, CGA, rutin, and vitamin C were the controls. The measured fluorescence values were expressed as a percentage of the control.

### 2.5. Determination of XOD-Inhibitory Activity

The inhibitory effect on XOD was spectrophotometrically determined according to a method by Arimboor et al. with some modifications [30]. Briefly, 0.7 mL of 0.2 XOD unit in sodium phosphate buffer (0.1 M; pH 7.5) and 0.1 mL of various concentrations of DMLEs (0.1–5.0 mg/mL), CGA, rutin, and ALP were mixed at 37°C for 5 min. The control did not contain test agents. After 5 min, 0.2 mL of 2 mM xanthine in distilled water was added to the mixture. Each mixture was shaken at 37°C for 15 min, and 1 mL of a stop buffer (1 N HCl) was added. ALP was used as a positive control. The absorbance of the mixture was measured at 290 nm using an ultraviolet-visible (UV-VIS) spectrophotometer (Optizen POP; CM Science, Busan, Korea). The IC_50_ values of the test samples and compounds were obtained using GraphPad Prism v.5.0 (GraphPad Software, Inc.).

### 2.6. Cell Cytotoxicity Assay

The human hepatocellular carcinoma cell line Hep3B (ATCC HB-8064) was grown in Dulbecco's modified Eagle's medium (DMEM; Merck KGaA, Darmstadt, Germany) supplemented with 10% (v/v) heat-inactivated fetal bovine serum (FBS), 100 U/mL penicillin, and 100 mg/mL streptomycin at 37°C under 5% CO_2_. Cell viability assay was examined following the procedure described earlier [20]. Briefly, cells (5 × 10^3^ cells/well) were plated onto a 96-well plate and cultured for 24 h at 37°C under 5% CO_2_. After 24 h, the cells were treated with various concentrations of DMLEs and incubated for 72 h. After 72 h, cells were washed with PBS, and 20 *µ*L of 3-(4,5-dimethylthiazol 2-yl)-2,5-diphenyltetrazolium bromide (MTT, 5 mg/mL) was added to each well. After incubation for 4 h, dimethyl sulfoxide (DMSO) was added to dissolve formazan crystals from MTT reduction, and the amount of formazan salt was determined by measuring the optical density at 540 nm using an ELISA plate reader (Bio-Rad, Hercules, CA, USA). By comparing the absorbance of the wells of cells treated with different concentrations of DMLEs with the control, the viability of cells after treatment with DMLEs was calculated. The concentration of DMLEs that reduced the cell viability by 50% (IC_50_) was recorded.

### 2.7. Animals

Sixty Sprague-Dawley rats (male, 5 weeks, 110–150 g) were purchased from Orient Bio (Seongnam, Korea) and randomly distributed into different experimental groups. The rats were housed in polypropylene cages at an ambient temperature of 25°C ± 1°C and 45%–55% relative humidity, with a 12 : 12-h light/dark cycle. Animals were provided with commercial food pellets and water ad libitum, unless stated otherwise. They were acclimatized to laboratory conditions for at least 1 week before experimenting on them. The experimental design was approved by the Institutional Animal Care and Use Committee of KemOn Inc. (Suwon, Korea) (Approval No. 2019-10-001), and all experiments were performed according to the guidelines established by the committee.

### 2.8. Induction of Hyperuricemia and Experimental Design

After acclimation for 7 d, the uricase inhibitor PO was intraperitoneally injected into rats to induce hyperuricemia. To study the antihyperuricemic effects of the 70% ethanol extract from DML (DMLE-70), the rats were randomly divided into six groups (*n* = 8 per group): (1) a normal control group (NC), (2) a PO-induced hyperuricemia model group (PC), (3) a PC + 50 mg/kg ALP group (ALT), (4) a PC + 50 mg/kg DMLE-70 group (DMEE-50), (5) a PC + 100 mg/kg DMLE-70 group (DMEE-100), and (6) a PC + 200 mg/kg DMLE-70 group (DMEE-200). Rats in all groups, except for NC, were injected intraperitoneally with 250 mg/kg PO prepared in 0.5% carboxymethylcellulose (CMC) with 0.1 M sodium acetate (pH 5.0) at 1, 3, 5, and 7 d after acclimation, and NC was treated with 0.5% CMC with 0.1 M sodium acetate. Rats of all groups, except for NC and PC, were administered extract once a day for 7 d from 1 h after the first injection of PO. The animals were anesthetized using isoflurane and sacrificed for sample collection.

### 2.9. Sample Collection and Analysis

Urine samples were collected during a 2-h period using a metabolic cage following the last substance administration. The volume of urine collected was 4–6 mL, and there was no difference in urine volume among the treated group. The samples were then centrifuged (3000 × g for 10 min at 4°C) to remove particulate contaminants, and the supernatants were stored at −80°C until analysis. After collecting urine samples, blood samples were collected via cardiac puncture and centrifuged (3000 × g for 10 min at 4°C) to obtain serum. The separated serum was stored at −80°C until analysis. According to the manufacturer's instructions, serum and urine levels of UA were determined using commercial assay kits (BioVision, Milpitas, CA, USA). At the end of the experiment, rats were euthanized by cervical dislocation after taking blood, and the liver tissue was collected and preserved at −20°C to determine the XOD activity.

### 2.10. Statistical Analyses

Data are expressed as the mean ± SEM. Statistical comparisons were analyzed by one-way analysis of variance (ANOVA) and Student's *t*-test using SPSS v.13.0. A *p*-value less than 0.05 was considered statistically significant.

## 3. Results

### 3.1. Extraction Yield

As a result of comparing the yield of DMLEs by the solvents, the yield of hot water extracts was highest at 24.14%, and the extraction yield decreased as the ethanol content increased (30% ethanol, 23.24%; 50% ethanol, 21.02%; and 70% ethanol, 19.66%).

### 3.2. Analysis of Chlorogenic Acid and Rutin Concentrations

**Figure 1:**
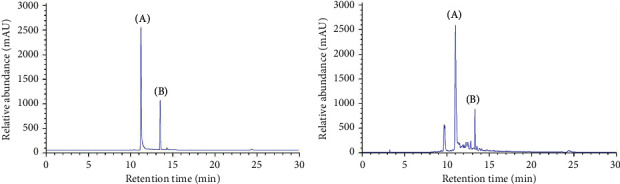
Representative liquid chromatogram of (A) standard and (B) *Dendropanax morbifera* leaf 70% ethanol extract.

Typical chromatograms of CGA and rutin are shown in Figure 1, representing the selected reaction monitoring chromatograms with a mass transition of CGA (*m/z* 355.00 ⟶ 145.15) and rutin (*m*/*z* 615.00 ⟶ 465.10). No interference peaks were detected at the retention times of all analytes. CGA and rutin were eluted at retention times of 11.321 and 13.502 min, respectively. In the LC-ESI-MS/MS analysis results, the concentrations of CGA and rutin increased depending on the ethanol concentration, and 70% DML ethanol extract (DMLE-70) had the highest CGA and rutin concentrations (Table 1).

### 3.3. Antioxidant Activity

DPPH radical scavenging activities were analyzed to determine the antioxidant activity of DMLEs. IC_50_ represents DPPH radical scavenging activities, and vitamin C was used as a positive control (Table 2). The IC_50_ of CGA was significantly low compared with that of DMLEs and rutin (*p* < 0.05). However, no significant difference in IC_50_ was observed between CGA and vitamin C. The IC_50_ of DMLE-70 was the most active among the DMLEs.

### 3.4. Measurement of ROS Production

The DCFH-DA assay was performed to evaluate the intracellular ROS scavenging activity of DMLEs and their active compounds in H_2_O_2_-treated AML-2/DX100 cells. After 400 *µ*M H_2_O_2_ treatment, ROS scavenging activity was not significant among DMLEs, CGA, rutin, and vitamin C at a 50 *µ*g/mL concentration. For treatment with 100 *µ*g/mL of DMLEs or compounds, the 50% ethanol extract showed the strongest ROS scavenging activity. There was no significant difference between the ROS scavenging activity of the water extract and DMLE-30. By contrast, there was a significant difference among all test materials, except for DMLE-30 and DMLE-70 (*p* < 0.05) (Table 3).

### 3.5. Determination of XOD-Inhibitory Activity

DMLEs and compounds of the XOD-inhibitory activity are shown in Table 4. The IC_50_ of ALP was 50 *µ*g/mL, which was the lowest among the test substances, followed by CGA. In addition, the IC_50_ of DMLE-70 was significantly low compared with that of other DMLEs (*p* < 0.05). However, the IC_50_ of rutin was more than 1000 *µ*g/mL, and there was almost no XOD-inhibitory activity.

### 3.6. Cell Cytotoxicity

In the MTT assay results, the IC_50_ values of DMLEs were more than 1.11 mg/mL (Table 5). The IC_50_ value of DMLW (2.08 mg/mL) was significantly high compared with that of other DMLEs (*p* < 0.05). In the ethanol extracts, the IC_50_ value decreased with an increase in the percentage of ethanol in the extraction solvents. However, no statistically significant difference was observed between each ethanol extract. Therefore, it was confirmed that the IC_50_ values of all DMLEs were more than 1.11 mg/mL, indicating almost no cytotoxicity.

### 3.7. UA Levels in Serum and Urine in the Hyperuricemic Rat Model

**Figure 2:**
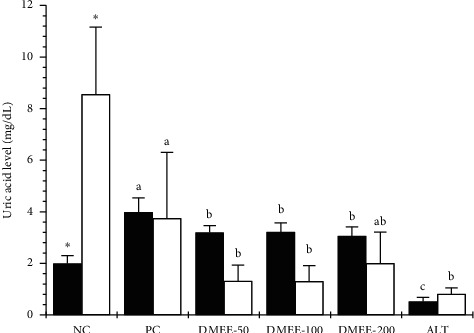
Effects of 70% ethanol extracts from *Dendropanax morbifera* leaf (DMLE-70) on serum (■) and urine uric acid (□) levels in rats with potassium oxonate-induced hyperuricemia. NC, normal control group; PC, PO-induced hyperuricemia model group; DMEE-50, PC + 50 mg/kg DMLE-70 group; DMEE-100, PC + 100 mg/kg DMLE-70 group; DMEE-200, PC + 200 mg/kg DMLE-70 group; ALT, PC + 50 mg/kg allopurinol group. ^*∗*^, significantly different from PC, *p* < 0.05. a, b, c, d: Mean values with different superscript letters in the same row show significant differences at *p* < 0.05 by one-way ANOVA.

The PC group exhibited significantly higher UA levels in serum and urine than the NC group did (*p* < 0.05), indicating that hyperuricemia was effectively established. UA levels in serum and urine were significantly reduced in all DMLE-70 and ALT groups compared with the PC group (*p* < 0.05) (Figure 2). In all DME groups, UA levels in urine were lower than those in serum. In PO-induced hyperuricemic rats, DMEE-200 reduced UA concentration in serum and increased UA excretion through the urine.

### 3.8. XOD Activity in Serum and Liver Tissue

XOD activity in serum showed no significant differences between all groups, except for the ALT group (Table 6). There was no significant difference in hepatic XOD activity between the PO-induced hyperuricemic rat group (PC) and the groups treated with DMLE-70 at concentrations of 50 and 100 mg/kg. However, hepatic XOD activity in the PC group was significantly increased compared with that in the group treated with DMLE-70 at a 200 mg/kg (*p* < 0.05) concentration. Meanwhile, treatment with ALP significantly reduced XOD activity in serum and liver tissue compared with that in all groups (*p* < 0.05).

## 4. Discussion

Hyperuricemia is a metabolic disorder characterized by a high UA level in serum due to an imbalance between UA production and excretion and causes gout [14]. Recently, the prevalence of hyperuricemia has continuously increased worldwide [31]. Currently, there are a limited number of drugs available for treating hyperuricemia, and many of these drugs have side effects.

Among the uricosuric agents, benzbromarone has been discontinued since 2003 because of severe hepatotoxicity reported in most European countries [32]. Although ALP belonging to XO inhibitors has been mainly used to treat hyperuricemia, this drug has been associated with severe cutaneous adverse reactions [33]. Therefore, many previous studies have been conducted to develop safe therapeutic agents using natural products for hyperuricemia without side effects [14, 19, 30].

This study showed the antioxidant, XOD-inhibitory, and antihyperuricemic activities of DMLEs and determined active components. The upregulation of XOD expression and activity increases reactive oxygen species (ROS) production. The XOD activity produces superoxide anions that can rapidly respond with nitric oxide to form the cytotoxic oxidant leading to various diseases including endothelial dysfunction and cardiovascular diseases [34]. When the local levels of ROS are increasing, they cause considerable cellular damage and generate other more reactive radicals [35]. As regards the production of ROS by XO, under conditions of oxidative stress, XO activity prevails to XDH activity, resulting in further ROS production. In cultures of endothelial cells, NOX maintains XO levels and XO is responsible for increased ROS production [36]. In this study, DMLE-70 had the strongest DPPH free radical scavenging activity and XOD-inhibitory activity among the DMLEs (Tables 2–4). These findings suggest that DMLEs inhibit hyperuricemia by inhibiting the generation of ROS through its XOD-inhibitory effect. However, DMLE-50 showed the strongest ROS scavenging activity at a 100 *µ*g/mL concentration among the DMLEs. On the basis of these results, DMLE-70 was selected for further *in vivo* experiments.

In the LC-MS/MS analysis results, DMLE-70 had the highest CGA and rutin concentrations (Table 1). CGA is an abundant polyphenol in human diet and Chinese medicines and possesses extensive pharmacological antimicrobial, antioxidant, anti-inflammatory, protection of cardiovascular and cerebrovascular system [37], and antigout activities [38]. Rutin is an essential flavonoid in herbal foods, which significantly reduced SUA levels in mice with hyperuricemia [39] and increased urinary UA concentration in rats with renal dysfunction [40]. In a previous study [41], the CGA and rutin contents from DML extracts in 70% ethanol for 2 h were the highest at 12.33 and 14.09 mg/g, respectively. In another study, the CGA concentration in the 30% DML ethanol extract collected in November was the highest at 36.55 mg/g, and the rutin content in the 60% DML ethanol extract collected in May was the highest at 116.71 mg/g [42]. In this study, the concentrations of CGA and rutin were approximately 0.5 and 0.25 times lower than those of Youn et al. but twice as high as those of Hwang et al. [41] (Table 1). This showed that the difference in CGA content and rutin depends on the harvesting location, season, and extraction conditions of DML [42].

We investigated whether DMLE extract showed antihyperuricemic effects in PO-induced hyperuricemic rats. In this study, DMEE-50, 100, and 200 significantly decreased serum UA levels compared with the PC group (*p* < 0.05). Additionally, DMEE-50 and 100 reduced UA levels in urine compared with the PC group (*p* < 0.05). Meanwhile, the urine UA levels of DMEE-200 were higher than those of DMEE-50 and 100, although there were no significant differences in urine UA levels between DMEE-200 and DMEE-50 and 100. These results suggest that DMEE-200 alleviates hyperuricemia by decreasing the UA concentration in the blood and increasing UA excretion in the urine (Figure 2).

Additionally, we demonstrated that DMLE could decrease serum UA levels in PO-induced hyperuricemic mice by inhibiting hepatic XOD activity. Inhibiting XOD activity in the liver reduces UA production and enhances UA excretion [43, 44]. DMLE-70 at all doses did not exhibit potent hypouricemic effects in serum compared with PC, but inhibiting hepatic XOD activity was significantly effective with DMEE-200 compared with the PC (Table 6), although the amount of uric acid excreted in urine was decreased compared with PC. These results elucidated that the hypouricemic effect of DMLE is caused by inhibiting XOD, which is a crucial enzyme in the biosynthetic pathway of UA. The effect of DMLE on renal transporters, which contribute to UA reabsorption, is unknown and will be the focus of our subsequent study.

This study demonstrated that DMLE reduced serum UA levels and enhanced the excretion of UA in rats with PO-induced hyperuricemia. DMLE has an antioxidant activity *in vitro* and inhibits XO activity of the liver and serum *in vivo*, thus preventing hyperuricemia. This study elucidates that DMLE exhibits beneficial effects and may be potentially helpful in treating hyperuricemia and gout.

## Figures and Tables

**Table 1:** Chlorogenic acid and rutin contents in *Dendropanax morbifera* leaf extracts by water and various ethanol concentrations.

Compounds	Chlorogenic acid (mg/g)	Rutin (mg/g)
DMLW	6.42 ± 0.02^a^	10.39 ± 0.06^a^
DMLE-30	13.25 ± 0.24^b^	16.39 ± 0.22^b^
DMLE-50	17.42 ± 0.30^c^	23.74 ± 0.31^c^
DMLE-70	21.46 ± 0.12^d^	29.52 ± 0.13^d^

DMLW, water extract of *Dendropanax morbifera* leaf; DMLE-30, 30% ethanol extract of *Dendropanax morbifera* leaf; DMLE-50, 50% ethanol extract of *Dendropanax morbifera* leaf; DMLE-70, 70% ethanol extract of *Dendropanax morbifera* leaf. ^a,b,c,d^Mean values with different superscript letters in the same row show significant differences at *p* < 0.05 by one-way ANOVA.

**Table 2:** 2,2-diphenyl-1-picryl-hydrazyl radical scavenging ability of *Dendropanax morbifera* leaf extracts and its active compounds.

Compounds	IC_50_ (*µ*g/mL)^1^	Relative activity (%)^2^
DMLW	102.53 ± 3.59^a^	5.36
DMLE-30	89.23 ± 2.36^b^	6.16
DMLE-50	85.13 ± 3.13^b^	6.46
DMLE-70	78.93 ± 0.58^c^	6.97
CGA	3.94 ± 0.05^d^	139.48
Rutin	37.60 ± 342^e^	14.63
Vitamin C	5.50 ± 0.40^d^	100.00

DMLW, water extract of *Dendropanax morbifera* leaf; DMLE-30, 30% ethanol extract of *Dendropanax morbifera* leaf; DMLE-50, 50% ethanol extract of *Dendropanax morbifera* leaf; DMLE-70, 70% ethanol extract of *Dendropanax morbifera* leaf; CGA, chlorogenic acid. ^1^IC_50_ value is a concentration of each sample for scavenging activity of 50% DPPH radical. ^2^A ratio of IC_50_ value compared with vitamin C used as a positive control. ^a,b,c,d^Mean values with different superscript letters in the same row show significant differences at *p* < 0.05 by one-way ANOVA.

**Table 3:** Reactive oxygen species (ROS) scavenging ability of *Dendropanax morbifera* leaf extracts and its active compounds.

Compounds	ROS scavenging abilities (%)	
	50 *µ*g/mL	100 *µ*g/mL
DMLW	42.74 ± 0.96	78.19 ± 2.43^a^
DMLE-30	43.22 ± 1.75	80.92 ± 3.01^ab^
DMLE-50	44.58 ± 2.82	84.86 ± 2.63^b^
DMLE-70	44.49 ± 3.31	57.14 ± 6.13^c^
CGA	41.67 ± 3.39	64.85 ± 3.31^d^
Rutin	44.58 ± 2.14	66.37 ± 2.72^d^
Vitamin C	47.50 ± 3.69	56.53 ± 4.08^c^

DMLW, water extract of *Dendropanax morbifera* leaf; DMLE-30, 30% ethanol extract of *Dendropanax morbifera* leaf; DMLE-50, 50% ethanol extract of *Dendropanax morbifera* leaf; DMLE-70, 70% ethanol extract of *Dendropanax morbifera* leaf; CGA, chlorogenic acid. ^a,b,c,d^Mean values with different superscript letters in the same row show significant differences at *p* < 0.05 by one-way ANOVA.

**Table 4:** Xanthine oxidase inhibition activity of *Dendropanax morbifera* leaf extracts and its active compounds.

Compounds	IC_50_ (*µ*g/mL)^1^	Relative activity (%)^2^
DMLW	527.07 ± 10.25^a^	1.95
DMLE-30	441.90 ± 5.15^b^	2.32
DMLE-50	324.29 ± 1.08^c^	3.16
DMLE-70	216.98 ± 7.41^d^	4.73
CGA	69.07 ± 2.85^e^	14.85
Rutin	>1000	—^3^
ALP	10.25 ± 0.26^f^	100.00

DMLW, water extract of *Dendropanax morbifera* leaf; DMLE-30, 30% ethanol extract of *Dendropanax morbifera* leaf; DMLE-50, 50% ethanol extract of *Dendropanax morbifera* leaf; DMLE-70, 70% ethanol extract of *Dendropanax morbifera* leaf; CGA, chlorogenic acid; ALP, allopurinol. ^1^The concentration of each compound needed to inhibit 50% of xanthine oxidase activity. ^2^A ratio of IC_50_ value compared with that of allopurinol. ^3^Not identified. ^a,b,c,d^Mean values with different superscript letters in the same row show significant differences at *p* < 0.05 by one-way ANOVA.

**Table 5:** Half maximal inhibitory concentration (IC_50_) of *Dendropanax morbifera* leaf extracts in the Hep3B cell lines after 72 h of exposure.

Compounds	IC_50_ (mg/mL)
DMLW	2.08 ± 0.16^a^
DMLE-30	1.23 ± 0.01^b^
DMLE-50	1.19 ± 0.02^b^
DMLE-70	1.11 ± 0.01^b^

DMLW, water extract of *Dendropanax morbifera* leaf; DMLE-30, 30% ethanol extract of *Dendropanax morbifera* leaf; DMLE-50, 50% ethanol extract of *Dendropanax morbifera* leaf; DMLE-70, 70% ethanol extract of *Dendropanax morbifera* leaf. ^a,b,c,d^Mean values with different superscript letters in the same row show significant differences at *p* < 0.05 by one-way ANOVA.

**Table 6:** Xanthine oxidase activity of *Dendropanax morbifera* leaf extracts in serum and liver tissue of the hyperuricemic rat model.

Compounds	Serum (mg/dL)	Liver (mU/mg protein)
NC	1.03 ± 0.05^a^	0.049 ± 0.003^a^
PC	0.96 ± 0.06^a^	0.046 ± 0.005^ab^
DMEE-50	1.00 ± 0.04^a^	0.043 ± 0.002^b^
DMEE-100	0.94 ± 0.03^a^	0.043 ± 0.002^b^
DMEE-200	0.94 ± 0.07^a^	0.037 ± 0.002^c^
Allopurinol	0.35 ± 0.01^b^	0.024 ± 0.001^d^

NC, normal control group; PC, PO-induced hyperuricemia model group; DMEE-50, PC + 50 mg/kg DMLE-70 group; DMEE-100, PC + 100 mg/kg DMLE-70 group; DMEE-200, PC + 200 mg/kg DMLE-70 group.^a,b,c,d^Mean values with different superscript letters in the same row show significant differences at *p* < 0.05 by one-way ANOVA.

## Data Availability

The data are available from the corresponding author upon request.
